# Fish polar lipids retard atherosclerosis in rabbits by down-regulating PAF biosynthesis and up-regulating PAF catabolism

**DOI:** 10.1186/1476-511X-10-213

**Published:** 2011-11-16

**Authors:** Constantina Nasopoulou, Alexandros B Tsoupras, Haralabos C Karantonis, Constantinos A Demopoulos, Ioannis Zabetakis

**Affiliations:** 1 Laboratory of Food Chemistry, Faculty of Chemistry, School of Sciences, National and Kapodistrian University of Athens, Panepistimioupolis of Zografou, 15771, Athens, Greece; 2 Laboratory of Biochemistry, Faculty of Chemistry, School of Sciences, National and Kapodistrian University of Athens, Panepistimioupolis of Zografou, 15771, Athens, Greece; 3 Department of Food and Nutrition Sciences, University of the Aegean, 2 Metropoliti Ioakim,814 00 Myrina, Lemnos, Greece

**Keywords:** Fish polar lipids, Mediterranean Diet, Cholesterol; Platelet Activating Factor (PAF), PAF Enzymes, Atherosclerosis

## Abstract

**Background:**

Platelet activating factor (PAF) has been proposed as a key factor and initial trigger in atherosclerosis. Recently, a modulation of PAF metabolism by bioactive food constituents has been suggested. In this study we investigated the effect of fish polar lipid consumption on PAF metabolism.

**Results:**

The specific activities of four PAF metabolic enzymes; in leukocytes, platelets and plasma, and PAF concentration; either in blood cells or plasma were determined. Samples were acquired at the beginning and at the end of a previously conducted study in male New Zealand white rabbits that were fed for 45 days with atherogenic diet supplemented (group-B, n = 6) or not (group-A, n = 6) with gilthead sea bream (*Sparus aurata*) polar lipids.

The specific activity of PAF-Acetylhydrolase (PAF-AH); a catabolic enzyme of PAF, was decreased in rabbits' platelets of both A and B groups and in rabbits' leukocytes of group A (p < 0.05). On the other hand the specific activity of Lipoprotein-associated Phospholipase A2 (Lp-PLA2); the catabolic enzyme of PAF in plasma was increased in both A and B groups in both leukocytes and platelets (p < 0.05). PAF-cholinephosphotransferase (PAF-CPT); a biosynthetic enzyme of PAF showed increased specific activity only in rabbits' leukocytes of group A (p < 0.05). Neither of the two groups showed any change in Lyso-PAF-acetyltransferase (Lyso-PAF-AT) specific activity (p > 0.05). Free and bound PAF levels increased in group A while decreased in group B (p < 0.05).

**Conclusions:**

Gilthead sea bream (*Sparus aurata*) polar lipids modulate PAF metabolism upon atherosclerotic conditions in rabbits leading to lower PAF levels and activity in blood of rabbits with reduced early atherosclerotic lesions compared to control group.

## Background

Platelet Activating Factor (PAF) [1] has been proposed as key factor in atherosclerosis development [2]. Dysregulation of PAF metabolism, lead to increased PAF-levels and triggers local inflammatory response onto the endothelium of arteries [2] with prominent role in atherogenesis [3,4].

The biosynthesis of PAF is accomplished through two distinctive enzymatic pathways; the *de novo *pathway; catalyzed by a specific dithiothreitol-insensitive CDP-choline: 1-alkyl-2-acetyl-sn-glycerol cholinephosphotransferase (PAF-cholinephosphotransferase; PAF-CPT, EC 2.7.8.16) that converts 1-O-alkyl-2-acetyl-glycerol to PAF and the remodeling one; catalyzed by lyso-PAF:acetyl-CoA acetyltransferase (Lyso-PAF-acetyltransferase; Lyso-PAF-AT, EC 2.3.1.67) [5] which acetylates lyso-PAF. On the other hand the catabolism of PAF in blood is catalyzed by a PAF-specific acetylhydrolase (PAF-AH, EC 3.1.1.47) whose plasma form is known as Lipoprotein-associated phospholipase A_2 _(Lp-PLA_2_) [6]. PAF-AH cleaves short chain acyl chains at the sn-2 position of phospholipids such as, oxidized phospholipids and PAF.

Various foodstuffs contain molecules that inhibit *in vitro *the activity of PAF [5]. Many micro- and macro-nutrients of the Mediterranean diet slow down the development of atherosclerosis [2,7]. In previous studies we have published that lipid minor constituents that inhibit *in vitro *PAF-induced thrombosis have been isolated from milk and yogurt, red and white wine and must, seed oils, olive oil, olive pomace and fish [2]. Recently we have published that supplementation of polar lipids from cultured gilthead sea bream (*Sparus aurata*) (GSBPL) inhibit early atherosclerosis development in diet induced hypercholesterolemic rabbits [8].

The aim of the study was to investigate how the consumption of GSBPL affects the enzyme activities of PAF metabolism and PAF levels in rabbits' blood cells and plasma upon atherosclerotic conditions.

## Results

### Isolation of fish polar lipids

Gilthead sea bream polar lipids (GSBPL) constituted the 0.23% of the whole fresh fish weight, since an amount of 28.0 g of polar lipids were isolated from 12.0 kg of cultured gilthead sea bream (*S. aurata*).

### Liquid chromatographic analysis

**Figure 1:**
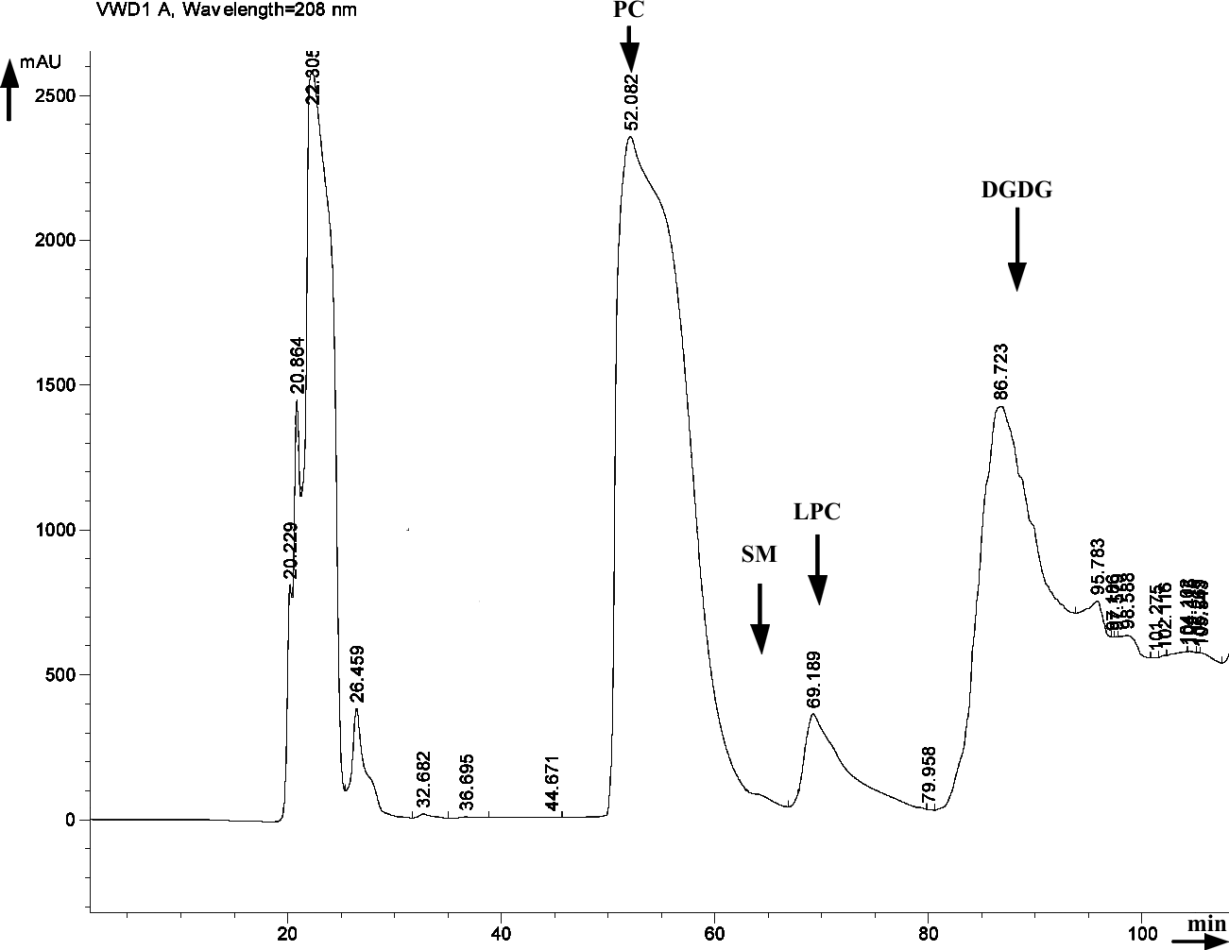
**Representative HPLC chromatograph of GSBPL**. Mobile phase was composed of solvent A; acetonitrile/methanol (60:40 v/v), solvent B; methanol (100%) and solvent C; water (100%). The mobile phase flow rate was adjusted to 1.0 mL/min, and UV detection wavelength was set at 208 nm. After 55 min holding of 100% solvent A, a linear ramp up to 100% B over 5 min was developed, followed by a 10 min holding of 100% B, then a linear ramp up to 100% C over 5 min was followed by a 30 min holding of 100% C. A 35 min equilibration was performed before the next analysis. Elution times of standard polar lipids were the following: phospahtidylcholine (PC) at 50-60 min, sphingomyelin (SM) at 62-67 min, lysophospahtidylcholine (LPC) at 68-80 min and digalactosyldiglycerides DGDG at 81-94 min.

A representative HPLC chromatographic profile of GSBPL is illustrated in Figure 1.

### Chemical determinations

The fraction of GSBPL that was eluted at 95-105 min upon HPLC separation exerted antagonistic effect against PAF toward washed rabbit platelets (data not shown) and in an effort to characterize qualitatively this fraction phosphorus and sugar determinations were performed. Phosphorus determination in this fraction was negative whereas sugar determination was positive giving 50.44 mmol of sugars per mg of fish polar lipid fraction expressed as glucose.

### Gas chromatographic analysis

The results of esterified fatty acids analysis in GSBPL by gas chromatography are presented in Table 1. Quantitatively the most abundant fatty acids identified in GSBPL were palmitic acid (16:0), oleic acid (18:1; cis Δ9), eicosapentaenoic acid (20:5; cis, cis, cis, cis, cis-Δ5,Δ8,Δ11,Δ14,Δ17) and docosahexaenoic acid (22:6; cis, cis, cis, cis, cis, cis-Δ4,Δ7,Δ10,Δ13,Δ16,Δ19).

**Table 1:** Gas chromatographic analysis of fatty acids in Gilthead Sea Bream Polar Lipids (GSBPL).

Fatty acid	Concentration (ppm)
14:0	17.0 ± 3.40
16:0	419 ± 83.9^a^
16:1 (omega-7)	30.6 ± 6.12
18:0	99.8 ± 20.0
18:1 cis (omega-9)	155 ± 77.7^a^
18:1 trans (omega-9)	29.4 ± 5.88
18:2 (omega-6)	62.1 ± 12.4
18:3 (omega-3)	Traces
20:4 (omega-6)	18.8 ± 3.77
20:5 (omega-3)^b^	152 ± 30.6^a^
22:5 (omega-3)	26.7 ± 5.33
22:6 (omega-3)^c^	405 ± 80.9^a^

^a^: Denotes quantitatively the most abundant fatty acids identified in GSBPL; ^b^: Eicosapentaenoic acid; EPA; ^c^: Docosahexaenoic acid; DHA).

### PAF-basic metabolic enzyme specific activities

The PAF-metabolic specific enzyme activities of the two experimental groups A and B at the beginning and at the end of the experimental diet are presented in Table 2. Specific enzyme activities and PAF levels of both A and B groups were comparable at the beginning of the study (p > 0.05). In group A, specific activity of PAF-CPT in leukocytes was significantly increased after 45 days on atherogenic diet compared to baseline (p < 0.05). In contrast, in group B PAF-CPT specific activity in Leukocytes did not change compared to its initial values. The increase of PAF-CPT specific activity in group A resulted in higher values in group A compared to group B at the end of the study (p < 0.05). The specific activity of PAF-CPT in platelets of group A, unlike of its activity in leukocytes, was comparable to its initial values after 45 days. On the other hand PAF-CPT specific activity in platelets of group B was significantly decreased (p < 0.05), but values of PAF-CPT specific activity of both A and B groups at the end of the study were not statistically different.

The specific activity of Lyso-PAF-AT in leukocytes in both A and B groups were not statistically different at the end of the study compared to baseline. The increase of Lyso-PAF-AT activity in leukocytes of group A failed to reach statistical significance, but after 45 days this activity was higher compared to that of group B (p < 0.05). In platelets of group A the specific activity of Lyso-PAF-AT was not statistically different after 45 days, whereas in platelets of group B, this enzyme activity was significantly reduced (p < 0.05) but values between A and B groups at 45 days were not statistical different.

The specific activity of PAF-AH in leukocytes of group A was significantly reduced at 45 days compared to baseline values (p < 0.05). In contrast, PAF-AH specific activity in leukocytes of group B was significantly increased compared to baseline (p < 0.05). PAF-AH specific activity in platelets in both A and B groups was significantly reduced at the end of the study (p < 0.05). In contrast to PAF-AH in platelets, specific activity of Lp-PLA_2 _in rabbits' plasma of both A and B groups was significantly increased (p < 0.05) after 45 days compared to baseline.

**Table 2:** PAF metabolic enzyme specific activities in plasma, leukocytes and platelets along with PAF levels in rabbit blood upon experimental diet.

Biochemical parameter	Time (days)	A (n = 6)	B(n = 6)
PAF-CPT specific activity in Leukocytes (nmol PAF/min/mg protein)	0	0.85 ± 0.29	0.57 ± 0.36
	45	2.52 ± 0.81^a^	0.66 ± 0.51^b^
PAF-CPT specific activity in platelets (nmol PAF/min/mg protein)	0	2.04 ± 1.11	1.49 ± 0.80
	45	1.89 ± 1.30	0.49 ± 0.31^a^
Lyso PAF AT specific activity in Leukocytes (nmol PAF/min/mg protein)	0	0.0545 ± 0.0427	0.0200 ± 0.0149
	45	0.1102 ± 0.0517	0.0256 ± 0.0239^b^
Lyso PAF AT specific activity in platelets (nmol PAF/min/mg protein)	0	0.0047 ± 0.0023	0.0042 ± 0.0030
	45	0.0029 ± 0.0022	0.0011 ± 0.0006^a^
PAFAH specific activity in Leukocytes (nmol PAF/min/mg protein)	0	340 ± 61.3	273 ± 109
	45	163 ± 15.7^a^	581 ± 340^b^
PAFAH specific activity in Platelets (nmol PAF/min/mg protein)	0	96.5 ± 30.4	112 ± 20.2
	45	25.0 ± 7.62^a^	30.8 ± 10.7^a^
Lp-PLA2 specific activity in plasma (pmol PAF/min/μL PPP)	0	128 ± 35.3	109 ± 15.2
	45	174 ± 21.6^a^	239 ± 56.7^a^
PAF levels in plasma; Free PAF levels (pM)	0	3.1 ± 1.7	3.04 ± 1.23
	45	5.0 ± 1.8^a^	0.24 ± 0.12^a,b^
PAF levels in cells; Bound PAF levels (pM)	0	0.88 ± 0.15	1.08 ± 0.36
	45	1.8 ± 0.34^a^	0.18 ± 0.08^a,b^

Group A followed a 1% cholesterol atherogenic diet, while group B followed the atherogenic diet supplemented with 0,06% (w/w) Gilthead Sea Bream (*Sparus aurata*) Polar Lipids (GSBPL). Results are expressed as Mean Values ± Standard Deviation. ^a^: Denotes statistically significant difference within the same group (day 45 vs day 0; p < 0.05), according to paired t-test. ^b^: Denotes statistically significant difference between groups A and B (at 0 or 45 day; p < 0.05), according to one-way ANOVA test.

### PAF levels in rabbits' blood

Free and bound PAF levels in both A and B groups were comparable at the beginning of the study (p > 0.05). At the end of the study both free and bound PAF levels were statistically higher in group A (p < 0.05) and lower in group B (p < 0.05) compared to the initial values, leading to higher values in group A compared to group B (p < 0,05) (Table 2).

## Discussion

Atherosclerosis still remains a leading cause of morbidity and mortality world-wide. PAF and its metabolism have been proposed as key factors in atherosclerosis development [2-4,9]. In a research effort it has been shown that many food-derived bioactive compounds alter the metabolism and/or the activity of inflammatory mediators like PAF both in *in vitro *and *in vivo *experiments, contributing in a favorable clinical profile when consumed in the diet [5].

Epidemiological and clinical trials have shown a beneficial effect of fish consumption toward cardiovascular diseases [10-12], attributed mainly to omega-3 fatty acids [12-14]. However, recently we showed that beyond omega-3 fatty acids, the consumption of GSBPL led to an inhibition of early atherosclerosis development in diet induced hypercholesterolemic rabbits [8]. In the present study whole blood, plasma, leukocytes and platelets of that previously conducted study [8] were used in order to investigate how the consumption of GSBPL upon atherosclrerotic conditions affect the activities of PAF metabolic enzymes and PAF levels in rabbits. Leukocytes and platelets were chosen for this study due to their critical role in the onset of atherosclerosis [2,15,16].

The low content of cultured gilthead sea bream (*Sparus aurata*) in polar lipids (0.23% of the whole fresh fish weight) indicates their high antiatherogenic activities since a 75% retardation of early atherosclerosis development has been observed compared to control group as a result of 133 mg consumption of GSBPL per rabbit per day as noted previously [8]. Moreover, HPLC chromatographic behavior, chemical determinations and fatty acid analysis in GSBPL shows that the bioactive polar lipids are peculiar glycolipids rather than classic phospholipids with palmitic, docosahexaenoic (DHA), oleic and eicosapentaenoic (EPA) fatty acids mainly esterified in their structures.

In the present study leukocytes and platelets in rabbits upon atherosclerotic conditions have been shown to contribute to an increased PAF biosynthesis. The increased PAF biosynthesis in group A comes from an increased activity of PAF-CPT in leukocytes along with a decreased activity of PAF-AH in both leucocytes and platelets. The increased *de novo *biosynthesis and the decreased catabolism of PAF in leukocytes and platelets are in accordance with the elevated PAF levels in both plasma (free PAF) and cells (bound PAF). These results are paralleled with both the formation of early atherosclerosis lesions and the increased aggregatory response of platelets toward PAF; expressed as reduced EC_50 _that had been previously observed in group, which was not administered with fish polar lipids [8]. These observations are in accordance with the fact that, on one hand, elevated PAF levels can trigger platelet stimulation and local inflammatory response onto endothelium [2,4,17], while, on the other hand, PAF-CPT activity in leukocytes is positively correlated with increased inflammation [18].

Atherogenic diet supplemented with GSBPL in group B has previously shown statistically significant lower early atherosclerotic lesions compared to group A [8]. In the present study, this effect was paralleled by a modulation in PAF metabolism in leukocytes and platelets of group B compared to that of group A. In contrast to group A, PAF-CPT in leukocytes of group B ceases further increase upon atherosclerotic conditions. Moreover a decrease of both PAF-CPT and Lyso PAF-AT activities were shown in platelets of group B. As a response of reduced PAF biosynthesis in platelets, platelet PAF-AH was also decreased; an observation consistent with data indicating that intracellular PAF acetylhydrolase activity is a critical component of stimulated PAF production [19]. The reduced PAF levels in both plasma and cells are in accordance with the observed reduction of PAF biosynthetic enzymes activities in platelets and the increased activity of Lp-PLA2 in plasma. These observations are also in agreement with the previous notion that platelets are less sensitive to aggregation towards PAF in group administered with fish polar lipids, where increased EC_50 _values were observed [8]. The increased activity of Lp-PLA2 in plasma of both A and B group showed a response of this enzyme towards atherogenic conditions independently of the GSBPL co-supplementation that cannot be predicted only by PAF metabolism modulation in leukocytes and platelets.

## Conclusions

We found that GSBPL modulate PAF metabolic enzymes upon atherosclerosis in a way that PAF levels are maintained low. The modulation of PAF metabolic enzyme activities leads to reduced platelet aggregatory response toward PAF and reduced early atherosclerotic lesions in group consumed GSBPL [8]. The appreciation of the role of PAF and its metabolism in atherosclerosis provides a mechanistic framework for understanding and unravelling mechanisms where bioactive food micronutrients are implicated in atherogenesis prevention.

## Materials and methods

### Reagents

1-O-hexadecyl-2-[^3^H] acetyl-sn-glycerol-3-phosphocholine ([^3^H]-PAF) with a specific activity of 10 Ci/mmol was obtained from New England Nuclear & Dupont (Boston, MA, USA). 1-*O*-alkyl-2-*sn*-acetyl-glycerol (AAG) was purchased from BIOMOL International LP (Exeter, UK). Both 2,5-Diphenyloxazole (PPO) and 1,4-bis(5-phenyl-2-oxazolyl) benzene (POPOP) were purchased from BDH Chemicals (Dorset, UK). Liquid chromatography grade solvents and silica G for TLC were purchased from Merck (Darmstadt, Germany). All the rest analytical reagents and solvents were purchased from Sigma (St.Louis, MO, USA).

### Instruments

We performed HPLC analysis on a Hewlett-Packard series 1100 (Avondale, PA, USA), equipped with a G1314A HP UV spectrophotometer. A normal phase YMC-Pack HPLC column with amino functional groups 250 × 20 mm, S-5 μm (Whatman, Maidstone, UK) was used for the chromatographic profile of fish polar lipids, while a cation exchange column SCX Partisil HPLC column 250 × 4.6 mm, 10 μm (Whatman (Maidstone, UK) was used for PAF isolation.

We performed GC analysis on a Shimadzu CLASS-VP (GC-17A) gas chromatograph (Kyoto, Japan), equipped with a split/splitless injector, flame ionization detector and an Agilent J&W DB-23 fused silica capillary column 60 m × 0.251 mm, 0.25 μm, (Santa Clara, CA, USA).

Homogenizations were conducted at 40KHz with a VC50 supersonic sonicator (Newtown, CT, USA). The liquid scintillation counter used was a 1209 Rackbeta (Pharmacia, Wallac, Finland). PAF-induced platelet aggregation studies were performed in a model 400 VS aggregometer of Chrono-Log (Havertown, PA, USA) coupled to a Chrono-Log recorder at 37°C with constant stirring at 1200 rpm.

### Isolation of fish polar lipids

Fish polar lipids were extracted from cultured gilthead sea bream (*S. aurata*) cultivated in Nireus Aquaculture S.A., situated in Chios Island, Greece. Total lipids were extracted according to the Bligh-Dyer method [20] and were further separated into polar and neutral lipids by counter-current distribution [21]. GSBPL were stored at -20°C until further analysis.

### Chemical determinations in fish polar lipids

Phosphorus determination was carried out according to the method of Bartlett [22] and sugar determination was carried out according to the method of Galanos and Kapoulas [23].

### Fish polar lipid chromatographic profile

We acquired fish polar lipid chromatographic profile by HPLC on an amino functional group HPLC column using a modified previously described elution system [24].

### Gas Chromatographic analysis of fish polar lipids Fatty acids

We prepared fatty acid methyl esters of GSBPL using a solution 0.5N KOH in 90% aqueous CH_3_OH and then we extracted them by n-hexane. The fatty acid analysis was carried out using the internal standard method as previously described [25].

### Study design

At the beginning and after 45 days, of a previously conducted study [8], we collected blood from twelve male New Zealand white rabbits and we determined PAF-levels and isolated leukocytes, platelets and plasma where we determined the specific activities of the four PAF-basic metabolic enzymes; PAF-CPT, Lyso-PAF-AT, PAF-AH and Lp-PLA2. Animal handling and treatment have been conducted as previously described [8]. In that study, twelve male New Zealand white rabbits of same weight and age were randomly divided into two groups of six animals each and were given specific diets for 45 days. Group A was the control group and was given typical diet supplemented with 1% cholesterol as atherogenic diet, while Group B was given atherogenic diet enriched with 0.06% (w/w) GSBPL. The research protocols of the study have been performed with the approval of local veterinary authorities and animal ethics committee.

### Isolation of plasma, leukocytes and platelets from blood of rabbits

An amount of 9.0 mL of blood from hypercholesterolemic rabbits was obtained from each rabbit in 1.0 mL of an anticoagulant solution of 0.085 M sodium citrate/0.065 M citric acid, at the beginning (0 days) and at the end (45 days) of the study where isolation of plasma, leukocytes and platelets were performed as previously described [26]. In each case protein was determined according to the method of Bradford [27] with BSA as the protein standard.

### Determination of PAF-basic metabolic enzyme specific activities

We determined PAF metabolic enzyme specific activities in triplicate in aliquots of leukocyte and platelet homogenates or plasma prepared from blood of hypercholesterolemic rabbits taken at 0 and 45 days. PAF-CPT and Lyso-PAF-AT specific activities were determined as previously described [26,28]. Specific enzymes activities of PAF-CPT and Lyso-PAF-AT were expressed as nmol of produced PAF/min/mg of sample protein present in each assay. Specific activities of intracellular PAF-AH and Lp-PLA_2 _were determined as previously described by the TCA precipitation method [29]. We used aliquots of 2.0 μL of plasma to determine Lp-PLA_2 _specific activity and aliquots of leukocytes and platelet homogenates with a final concentration of 0.5 mg protein per mL per assay to determine PAF-CPT, Lyso-PAF-AT and intracellular PAF-AH specific activities.

### Determination of PAF levels in rabbit blood

Determination of free PAF levels (PAF levels in plasma) and bound PAF levels (PAF levels in blood cells) was carried out in 5.0 mL of blood as previously described [30,31].

### Statistical analysis

Normality of the data was examined by the Kolmogorov-Smirnov criterion before further analysis. Differences in enzyme specific activities and PAF levels within each group and between group A and B were conducted using the paired t-test and one way ANOVA test respectively. Data considered being statistically significant when p value was found below 0.05. The data were analyzed using a statistical software package (PASW 18 for Windows, SPSS Inc., Chicago, IL, USA).

## Abbreviations

PAF: Platelet Acticating Factor; PAF-CPT: PAF Cholinephosphotransferase; Lyso-PAF-AT: Lyso-PAF-Acetyltransferase; PAF-AH: PAF-Acetylhydrolase; Lp-PLA2: Lipoprotein associated phospholipase A2; GSBPL: gilthead sea bream polar lipids; EPA: Eicosapentaenoic acid; DHA: Docosahexaenoic acid.

## Competing interests

All authors declare that they have no competing interests.

## Authors' contributions

All authors read and approved the final manuscript. NC contributed to the design and coordination of the study, she performed the measurements, collected and analyzed the data and drafted the manuscript. TAB contributed to the conception and design of the study, he participated in the performance of the measurements and in the analysis of the data and he drafted the manuscript. HCK, DCA and ZI contributed to the conception and design of the study, and edited the manuscript.
